# Effects of acupuncture on nutritional state of patients with stable chronic obstructive pulmonary disease (COPD): re-analysis of COPD acupuncture trial, a randomized controlled trial

**DOI:** 10.1186/s12906-018-2341-3

**Published:** 2018-10-24

**Authors:** Masao Suzuki, Shigeo Muro, Motonari Fukui, Naoto Ishizaki, Susumu Sato, Tetsuhiro Shiota, Kazuo Endo, Tomoko Suzuki, Tadamichi Mitsuma, Michiaki Mishima, Toyohiro Hirai

**Affiliations:** 1 Department of Kampo Medicine, Aizu Medical Center, Fukushima Medical University School of Medicine, 21-2 Maeda, Tanisawa, Kawahigashi, Aizuwakamatsu, Fukushima 969-3492 Japan; 2 Department of Respiratory Medicine, Nara Medical University, 840 Shijo-Cho, Kashihara, Nara, 634-8521 Japan; 3 Respiratory Disease Center, Kitano Hospital, Tazuke Kofukai Medical Research Institute, 2-4-20 Ohgimachi, Kita-ku, Osaka, 530-8480 Japan; 4 Course of Acupuncture and Moxibustion, Faculty of Health Sciences, Tsukuba University of Technology, 4-12-7 Kasuga, Tsukuba, Ibaraki 305-8521 Japan; 5 Department of Respiratory Medicine, Graduate School of Medicine, Kyoto University, Yoshida, Konoe-cho, Sakyo-ku, Kyoto, 606-8501 Japan; 6 Department of Respiratory Medicine, Shiga General Hospital, 4-30 Moriyama-cho, Moriyama, Shiga 524-8524 Japan; 7 Department of Respiratory Medicine, Hyogo Prefectural Amagasaki General Medical Center, 2-17-77 Higashinanba-cho, Amagasaki, Hyogo 660-8550 Japan; 8 Noe Hospital, 1-3-25 Joto-ku, Osaka, 536-001 Japan

**Keywords:** COPD, Acupuncture, Nutritional state, Proinflammatory cytokine

## Abstract

**Background:**

There are an increasing number of evidences that chronic obstructive pulmonary disease (COPD) is a systemic illness and that bodyweight loss is its prominent manifestation. We focused on the nutritional outcomes to find out the effectiveness of acupuncture on nutritional state of COPD patients and on their prognosis in our previous interventional study.

**Methods:**

The present study is re-analysis of our previous interventional study, COPD Acupuncture Trial (CAT) published in 2012. Data from CAT was re-analyzed in terms of nutritional status, inflammatory biomarkers, and prognostic index. Nutritional states were evaluated by the measurements of body weight, body composition, and muscle strength, and the nutritional hematological examination results (retinol-binding protein (RBP), prealbumin (PA), transferrin (Tf), and hemoglobin (Hb) in serum), and inflammation biomarkers such as carboxyhemoglobin (COHb), High sensitivity C-reactive protein (Hs-CRP), Tumor Necrosis Factor-alpha (TNF-α), Interleukin 6 (IL-6), and Serum Amyloid A (SAA) were measured. The BODE index was measured in terms of prognosis. These measurements were compared between the real acupuncture group (RAG) and the placebo acupuncture group (PAG). All data are presented as mean (SD) or mean (95% CI). The difference between baseline and final volumes was compared using analysis of covariance (ANCOVA). Moreover, correlations between nutritional hematological examination scores and inflammation biomarker parameters were assessed using Spearman’s rank correlation coefficient.

**Results:**

After 12 weeks, the change in body weight was significantly greater in the RAG compared with the PAG (mean [SD] difference from baseline: 2.5 [0.4] in RAG vs − 0.5 [1.4] in PAG; mean difference between the groups: 3.00, 95% CI, 2.00 to 4.00 with ANCOVA). Patients in RAG also had improvements in the results of nutritional hematological examination (RBP, PA, Tf, Hb), Inflammation biomarkers (TNF-α, IL-6, SAA, Hs-CRP, COHb) and the BODE index.

**Conclusion:**

This study demonstrated some clear evidences that acupuncture can be a useful adjunctive therapy to improve nutritional state of COPD patients.

**Trial registration:**

UMIN Clinical Trials Registry (UMIN000001277). Retrospectively registered.

**Electronic supplementary material:**

The online version of this article (10.1186/s12906-018-2341-3) contains supplementary material, which is available to authorized users.

## Background

There are an increasing number of evidences that chronic obstructive pulmonary disease (COPD) is a systemic illness and that bodyweight loss is its prominent manifestation. Low body mass index (BMI) is closely associated with mortality in COPD [1]; Malnutrition occurs in approximately one-quarter to one-third of patients with moderate to severe COPD [2]; Weight loss may be the result of an increased imbalance in energy expenditure caused by inadequate intake of nutrient. Previous studies have suggested that underweight patients show higher metabolic rate (negative energy balance) [3], lower antioxidant capacity of skeletal muscles [4], and increased systemic inflammation [5], which may be causing weight loss and morbidity. The cachexia associated with COPD was traditionally believed to be more prevalent among those whose airflow limitation was due to predominant emphysema (the “pink puffer” hypothesis) [6].

Previous studies have also shown that patients with COPD exhibit low-grade systemic inflammation which is often associated with significant extra-pulmonary effects, such as cardiovascular abnormalities and skeletal muscle dysfunctions [7]. Gan et al. performed a systematic review of these studies and found that circulating leukocytes, fibrinogen, serum C-reactive protein (CRP) and tumor necrosis factor-alpha (TNF-α) levels were higher in COPD patients than in control subjects [8].

We previously demonstrated that dyspnea and exercise capacity evaluated with Borg scale scores and the 6-min walking distance (6MWD) were markedly improved with acupuncture in a prospective, randomized controlled trial in COPD patients who were receiving standard medication [9]. This study also showed some suggestive results that acupuncture was effective to improve nutritional state of patients.

Therefore, we re-analyzed the data focusing on the nutritional outcomes, inflammatory biomarkers, and oxidative stress which were not fully evaluated in our previous study, to find out the effectiveness of acupuncture on nutritional state of COPD patients and on their prognosis.

## Methods

### Study design and patients

This is re-analysis study of the data collected in the CAT (COPD Acupuncture Trial) published in 2012 [9]. The study is a randomized, single-blind, placebo-controlled, parallel group study which involved patients with moderate to very severe COPD. Patients with the diagnosis of COPD were eligible for inclusion of the study. All participants met the following criteria: 1) patients who had been diagnosed as stage II, III, or IV COPD, in accordance with the definition and criteria of the Global Initiative for Chronic Obstructive Lung Disease (GOLD) guidelines [10]; 2) patients who were clinically stable with no history of infections or exacerbation of respiratory symptoms, no changes in medication within 3 months preceding the study outset, and no signs of edema; 3) patients who were graded as stage II or higher on the Medical Research Council (MRC) criteria; 4) patients who were able to walk unassisted; 5) patients who had not been receiving pulmonary rehabilitation in the previous 6 months; and 6) outpatients. Patients presenting any evidence of cardiovascular disease, collagen disease, renal failure, thyroid dysfunction, hepatic function disorder, cancer, and severe mental disorders were excluded. This study was performed in accordance with the Declaration of Helsinki and its amendments, and the Guidelines for Good Clinical Practice for Epidemiological Studies and Clinical Research issued by the Japanese Ministry of Health. The Institutional Review Board of Tazuke Kofukai Foundation, Medical Research Institute, Kitano Hospital approved the study, and written informed consents were obtained from each patient. This study was registered with the UMIN Clinical Trials Registry (UMIN000001277).

### Intervention

Subjects in the real acupuncture group (RAG) received acupuncture treatment once a week for 12 weeks, in addition to daily medication. Selection of standardized acupuncture points was done in accordance with the past researches on acupuncture for pulmonary dysfunctions [11, 12] and literatures describing traditional prescription of acupuncture points for bronchial asthma and chronic bronchitis, of which the effect on COPD were verified through our clinical experiences over the past 10 years. Also, the importance of acupuncture points close to the respiratory accessory muscles was emphasized in the process of determination of the standardized treatment. The standardized acupuncture points used in the present study were: 1) Zhongfu (LU 1) and 2) Taiyuan (LU 9) in the lung meridian; 3) Futu (LI 18) in the large intestine meridian; 4) Guanyuan (CV 4) and 5) Zhongwan (CV 12) in the conception vessel; 6) Zusanli in stomach meridian 36 (ST 36); 7) Taixi (KI 3) in the kidney meridian; 8) Wangu in the gallbladder meridian (GB12); and 9) Feishu (BL 13), 10) Pishu (BL 20), and 11) Shenshu (BL 23) in the bladder meridian.

A Park sham device, which contains a needle (real or placebo), was used with a guide tube mounted on a base adherent to the skin [13]. The tip of the placebo needles used for the placebo acupuncture group (PAG) were blunt and appeared to be penetrating the skin but actually telescoped back into their handles. The real and placebo needles appear similar and of the same size (0.35 mm × 70 mm, stainless steel, Dong Bang Acupuncture Inc. Korea).

For RAG patients, needles were inserted to a depth ranging from 5 to 25 mm and manually rotated clockwise and counter-clockwise for 3–4 min at each point during 50-min treatment period. No electrical stimulation was performed. Perception of de qi during insertion and/or manipulation was confirmed at every point in the RAG.

The PAG underwent treatment at the same acupuncture points as the RAG. Perception of sensation during treatment sessions in PAG included pricking or poking but no sensation like de qi was reported.

### Outcomes

1. Main measurement of nutritional outcome is the change in body weight with Percent Ideal Body Weight (%IBW) of COPD patients after 12 weeks of acupuncture [14, 15]. %IBW was calculated with the following formulas; IBW = (height (m)) [2] × 22, %IBW = (real body weight / IBW) × 100 .
2. Prognosis outcome measurement is the BODE index, which is a multidimensional index that includes four factors that predict the risk of death: BMI (B); degree of air flow obstruction (O); functional dyspnea (D); and exercise capacity (E), assessed by the 6MWD [16].
3. Measurements of body composition are Midupper Arm Circumference (MAC), Triceps Skinfold (TSF) thickness, Scapula Skin folds (SSF) thickness and quadriceps circumference (above patellar 10 cm), all of which were measured except for Arm Muscle Circumference (AMC) which was calculated. Skinfold thickness was measured to assess changes in fat mass using Harpenden skinfold calipers (Holtain) according to its standard methodology [15]. Measurement was performed on the non-dominant hand, and in the case of paralysis, on the non-paralyzed side. Measurer evaluated the average value of the three measurements. The measurement of TSF was performed as follows. Subjects were in a lateral decubitus position, the upper arm was flexed by 90 ° at the elbow joint, a mark was placed at the midpoint between the shoulder bladder projection and the ulnar olecranon projection, the skin 1 cm away from the mark was picked up to so that the fat layer is separated from the muscle, and we measured the thickness by sandwiching the marked part with caliper. As for the measurement of SSF, with the patients in the lateral decubitus position, the same method as TSF was used at the portion of the shoulder blade lower corner. For MAC, the surroundings of the part marked for TSF was measured with a measuring tape. AMC was calculated by this formula: [AMC(cm) = AC-π × TSF(cm)]. As for the measurement of QC, patient was in supine position, a mark was put at 10 cm above from the upper edge of the patella toward the trunk of the non-dominant or non-paralyzed limb, and the peripheral diameter of the thigh at the mark was measured.
4. Muscle strength was evaluated as a hand grips, and the maximum inspiratory mouth pressure (MIP) and maximum expiratory mouth pressure (MEP) were measured using a standard mouthpiece and a device (Vitaropower KH115, Chest MI Co. Ltd., Tokyo, Japan) according to American Thoracic Society/European Respiratory Society [17].
5. Measurements of Nutritional Hematological Examination and Inflammation Biomarkers

Biochemical tests such as serum retinol-binding protein (RBP), pre-albumin (PA), transferrin (Tf) and hemoglobin (Hb) were measured as nutrition indices, and carboxyhemoglobin (COHb) within arterial blood gas, High sensitivity C-reactive protein (Hs-CRP), Tumor Necrosis Factor-alpha (TNF-α), Interleukin 6 (IL-6), and Serum Amyloid A (SAA) were measured as inflammatory biomarkers. Serum TNF-α, IL-6 and Hs-CRP were measured using commercially available enzyme-linked immunosorbent assay kits (Mitsubishi BCL, inc., Tokyo, Japan).

### Statistical analysis

All data are presented as means and SDs or 95% confidence intervals (CI). Analyses of outcome measures were performed according to the Full analysis set. The difference between baseline and final values was compared using analysis of covariance (ANCOVA) with baseline values and age as covariates, and treatment group as the factor of interest.

Correlations between nutritional examination scores and inflammation biomarker parameters were assessed by looking at their changes from baseline to post treatment for each group. Spearman’s rank correlation coefficient was calculated. The magnitude of the results was determined by calculating the effect size *d*. According to Cohen, *d* = 0.2 is a small treatment effect, *d* = 0.5 is a moderate effect, and *d* = 0.8 is a large effect [18].

All main analyses in the present study were carried out with JMP® 11 (SAS Institute Inc., Cary, NC, USA). In order to avoid bias, the biostatistician performed the statistical analysis with grouping information masked.

## Results

### Study population

During the period from July 2006 to March 2009, 111 COPD patients were found to meet the inclusion/exclusion criteria. Sixty-eight patients out of these 111 agreed to participate in the study. Of those participants, six were unable to complete the study because they felt it was too difficult to continue (two in the PAG and one in the RAG) and because of acute exacerbation due to respiratory infections (three in the RAG). Baseline characteristics of the patients in each group, are shown in Table 1. All medications remained unchanged through the study period.

**Table 1:** Baseline subject characteristics

	PAG (*n* = 34)	RAG (n = 34)	Mean difference
Sex (M/F)	32/2	31/3	
Age yr	72.5 [7.4]	72.7 [6.8]	0.2
MRC	2.9 [1.1]	3.3 [1.0]	0.4
Body Weight (kg)	56.0 [13.8]	54.6 [10.6]	−1.4
BMI (Kg/m^2^)	21.1 [3.9]	21.2 [3.9]	0.1
Brinkman Index	1433.9 [759.8]	1292.5 [526.4]	−141.4
GOLD criteria			
I	0	0	
II	13	6	
III	8	16	
IV	13	12	
	3.0 [0.9]	3.2 [0.7]	0.2
HOT	11	9	
Pulmonary function			
FVC (L)	3.0 [0.7]	2.8 [0.6]	−0.2
FEV_1_ (L)	1.2 [0.4]	1.0 [0.3]	−0.2
% FEV_1_(%)	48.0 [16.5]	44.5 [16.3]	−3.5

*PAG* placebo acupuncture group, *RAG* real acupuncture group, *MRC* medical research council, *BMI* body mass index, *GOLD* global initiative for chronic obstructive lung disease, *HOT* home oxygen therapy, *FVC* forced vital capacity, *FEV*_1_ forced expiratory volume in 1 second, % *FEV*_1_ forced expiratory volume in 1 second in predicted

Also, no patient had supplement or vitamin newly prescribed by the hospital during 6-month period prior to the study.

### Body weight

**Fig. 1:**
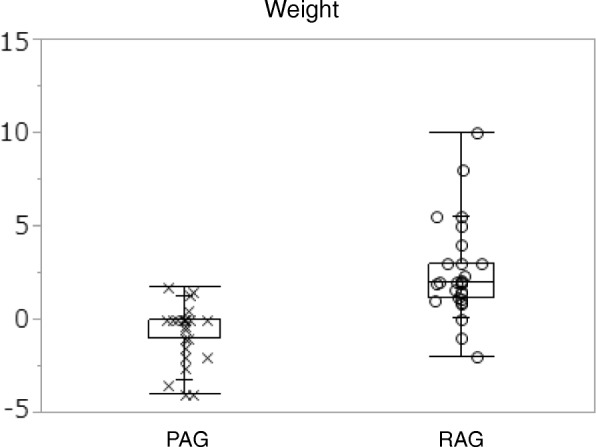
Difference of body weight between baseline and after 12 weeks for each group is shown. The difference in the body weight in the RAG (2.5 [0.4]) was statistically significant compared with that in the PAG (− 0.5 [1.4]) (mean difference 3.00, 95% CI 2.00 to 4.00 by ANCOVA). PAG; Placebo Acupuncture Group, RAG; Real Acupuncture Group

After 12 weeks of treatment, the body weight increased from 55.5 kg [10.4] to 58.0 kg [10.8] in the RAG. On the other hand, there was no change in the body weight in the PAG before and after the treatment (56.8 kg [13.8] to 56.3 kg [13.0], respectively). The difference in the body weight in the RAG (2.5 [0.4]) was statistically significant compared with that in the PAG (− 0.5 [1.4]) (mean difference 3.00, 95% CI 2.00 to 4.00 by ANCOVA) (Table 2, Fig. 1). Improvement in the %IBW found in the RAG was statistically significant compared to that in the PAG. The Cohen’s *d* effect size for the Body weight and %IBW were large(*d* = 2.87 and *d* = 1.51), respectively.

**Table 2:** Changes in the nutritional outcome and prognosis

	Baseline	After 12 weeks	Change from baseline to post treatment measurements	MD	*95% CI*	Effect size (*d*)
Main measurement of nutritional outcome						
Body Weight (kg)						
PAG (n 32)	56.8 [13.8]	56.3 [13.0]	−0.5 [1.4]	3.00	2.00, 4.00	2.87
RAG (n 30)	55.5 [10.4]	58.0 [10.8]	2.5 [0.4]			
%IBW: ideal body weight (%)						
PAG (n 32)	96.7 [17.7]	95.9 [16.5]	−0.8 [2.4]	5.20	3.52, 7.00	1.51
RAG (n 30)	98.6 [17.7]	103.0 [18.3]	4.4 [4.3]			
Prognosis outcome measurement						
BODE index						
PAG (n 32)	3.3 [2.4]	3.3 [2.4]	−0.03[1.0]	−1.17	−1.55, − 0.55	1.06
RAG (n 30)	3.9 [2.4]	2.7 [1.7]	−1.2 [1.2]			
BMI						
PAG (n 32)	0.5 [0.5]	0.4 [0.5]	−0.1 [1.3]	−0.11		
RAG (n 30)	0.4 [0.5]	0.2 [0.4]	−0.2 [0.5]			
%FEV_1_						
PAG (n 32)	1.5 [1.1]	1.7 [1.1]	0.2 [0.4]	−0.29		
RAG (n 30)	1.8 [1.0]	1.6 [1.0]	−0.1 [0.6]			
mMRC						
PAG (n 32)	0.9 [1.1]	0.8 [1.1]	−0.1[0.5]	−0.51		
RAG (n 30)	1.2 [1.0]	0.6 [0.8]	−0.6 [0.7]			
6MWD						
PAG (n 32)	0.4 [0.7]	0.4 [0.7]	0.0 [0.3]	−0.30		
RAG (n 30)	0.5 [0.9]	0.3 [0.5]	−0.3 [0.7]			

*MD* mean difference, %*IBW* ideal body weight (%), *BODE index* BMI (B); degree of air flow obstruction (O); functional dyspnea (D); and exercise capacity (E), assessed by the 6MWD. *BMI* body mass index, %*FEV*_1_ % forced expiratory volume in 1 s, *mMRC* Modified Medical Research Council, 6*MWD* 6 minute walk distance

### BODE index

**Fig. 2:**
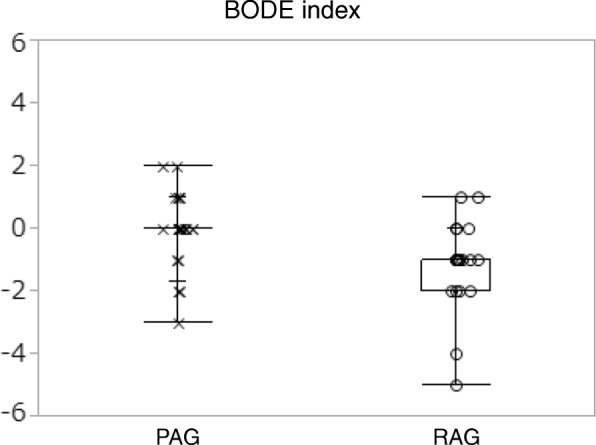
Difference of BODE index between baseline and after 12 weeks for each group is shown. The difference in the BODE index in the RAG (− 1.2 [1.2]) was statistically significant compared with that in the PAG (− 0.03 [1.0]) (mean difference − 1.17, 95% CI -1.55 to − 0.55 by ANCOVA). PAG; Placebo Acupuncture Group, RAG; Real Acupuncture Group

The BODE index improved from 3.90 (2.38) to 2.70 (1.73) in the RAG. In the PAG, on the other hand, there was no improvement in the BODE index before and after placebo acupuncture treatment (3.28 [2.40] and 3.28 [2.37], respectively). The difference in the BODE index in the RAG (− 1.2 [1.2]) was statistically significant compared with that in the PAG (− 0.03 [1.0]) (mean difference − 1.17, 95% CI -1.55 to − 0.55 by ANCOVA) (Table 2, Fig. 2). The Cohen’s *d* effect size for the BODE index was large(*d* = 1.06).

### Measurements of body composition and muscle strength

Significant improvements in the body composition (MAC, TSF, SSF, quadriceps circumference), and muscle strength (Grip strength, Respiratory muscle strength) were found in the RAG compared to those in the PAG. Only AMC showed no significant improvement (Table 3).

**Table 3:** Changes in the body composition and muscles strength

	Baseline	After 12 weeks	Change from baseline to post treatment measurements	MD	*95% CI*	*Effect size (d)*
Measurements of body composition						
MAC (cm)						
PAG (n 32)	24.3 [3.6]	24.0 [3.6]	− 0.3[1.1]	1.77	1.15, 2.39	1.42
RAG (n 30)	24.7 [3.4]	26.1 [3.9]	1.4 [1.3]			
TSF (mm)						
PAG (n 32)	11.3 [5.5]	9.9 [5.2]	−1.4 [2.5]	5.56	4.10, 7.03	1.92
RAG (n 30)	12.0 [4.9]	16.2 [6.2]	4.2 [3.3]			
AMC (cm)						
PAG (n 32)	20.9 [2.8]	21.0 [2.8]	0.1 [1.0]	0.10	−0.44, 0.64	0.10
RAG (n 30)	21.1 [2.6]	21.3 [2.9]	0.2 [1.1]			
SSF (mm)						
PAG (n 32)	13.4 [5.1]	12.8 [5.2]	−0.6 [2.8]	3.55	2.04, 5.05	1.22
RAG (n 30)	15.9 [5.9]	18.9 [6.3]	3.0 [3.1]			
QC (AP 10 cm) (cm)						
PAG (n 32)	38.3 [4.6]	38.1 [4.6]	−0.2 [0.7]	1.86	1.13, 2.59	1.34
RAG (n 30)	37.9 [4.2]	39.6 [4.6]	1.7 [1.9]			
Muscles strength						
Grip strength (Right) (kg)						
PAG (n 32)	28.9 [6.1]	28.6 [5.5]	−0.3 [2.3]	1.55	0.42, 2.67	0.73
RAG (n 30)	27.5 [6.2]	28.8 [5.9]	1.3 [2.1]			
Grip strength (Left) (kg)						
PAG (n 32)	28.4 [5.4]	27.4 [4.9]	−1.0 [2.5]	1.94	0.73, 3.14	0.85
RAG (n 30)	26.5 [6.4]	27.5 [6.6]	1.0 [2.2]			
Respiratory muscle strength MEP (H_2_Ocm)						
PAG (n 32)	63.5 [21.5]	61.8 [22.9]	−1.7 [12.2]	36.09	26.41, 45.77	1.90
RAG (n 30)	59.5 [21.6]	93.9 [32.2]	34.4 [24.3]			
MIP (H_2_Ocm)						
PAG (n 32)	56.4 [20.6]	55.4 [19.5]	−1.0 [11.9]	14.83	8.49, 21.16	1.19
RAG (n 30)	60.8 [20.6]	74.6 [15.8]	13.8 [13.0]			

*MD* mean difference, *MAC* midupper arm circumference, *TSF* triceps skinfolds, *AMC* arm muscle circumference, *SSF* scapula skinfolds, *QC* Quadriceps circumference, *MEP* maximum expiratory mouth pressure, *MIP* maximum inspiratory mouth pressure

### Nutritional hematological examination

Improvements in the nutritional hematological measurements (RBP, PA, Tf, Hg) found in the RAG were statistically significant compared to those in the PAG (Table 4).

**Table 4:** Changes in the Nutritional Hematological and Inflammation Biomarkers

	Baseline	After 12 weeks	Change from baseline to post treatment measurements	MD	*95% CI*	*Effect size (d)*
Nutritional Hematological						
RBP (mg/dL)						
PAG (n 20)	3.0 [0.7]	3.0 [0.6]	0.02 [0.5]	0.98	0.48, 1.46	1.60
RAG (n 22)	3.3 [1.5]	4.3 [1.3]	1.0 [0.7]			
PA (mg/dL)						
PAG (n 32)	23.4 [3.7]	22.9 [4.6]	−0.5 [2.6]	2.84	0.93, 4.76	0.77
RAG (n 30)	23.0 [4.9]	25.4 [6.7]	2.4 [4.7]			
Tf (mg/dL)						
PAG (n 20)	218.9 [76.6]	208.0 [67.9]	−10.9 [8.8]	54.1	34.53, 71.82	2.94
RAG (n 22)	216.4 [38.1]	259.6 [45.0]	43.2 [24.0]			
Hb (g/dL)						
PAG (n 32)	14.1 [1.1]	13.9 [1.2]	−0.2 [0.9]	0.90	0.54, 1.27	1.17
RAG (n 30)	13.9 [1.6]	14.6 [1.3]	0.7 [0.6]			
Inflammation Biomarkers						
Hs-CRP (ng/mL)						
PAG (n 12)	735.3 [445.6]	717.8 [396.6]	−17.4 [71.7]	− 576.58	− 610.60, −44.23	1.57
RAG (n 10)	1109.3 [437.2]	515.3 [247.6]	− 594.0 [542.4]			
TNF-α (pg/mL)						
PAG (n 12)	2.5 [0.9]	2.8 [1.0]	0.4 [0.8]	−2.09	−2.44, −0.42	1.56
RAG (n 10)	3.3 [1.5]	1.6 [1.2]	−1.7 [1.8]			
IL-6 (pg/mL)						
PAG (n 12)	2.9 [1.0]	3.6 [1.4]	0.7 [1.5]	−2.34	−3.13, −0.87	1.55
RAG (n 10)	3.3 [1.1]	1.7 [1.0]	−1.7 [1.6]			
SAA (μg/mL)						
PAG (n 12)	5.0 [1.7]	5.5 [2.4]	0.5 [1.0]	−2.10	−3.34, −1.02	1.75
RAG (n 10)	4.4 [1.7]	2.7 [0.8]	−1.6 [1.4]			
COHb (%)						
PAG (n 32)	1.4 [1.0]	1.9 [1.1]	0.5 [0.9]	−1.13	−1.51, −0.69	1.10
RAG (n 30)	1.4 [1.2]	0.8 [0.6]	−0.6 [1.1]			

*MD* mean difference, *RBP* Retinol-Binding Protein, *PA* Pre-Albumin, *Tf* Transferrin, *Hb* Hemoglobin, *Hs-CRP* High sensitivity C-reactive protein, *TNF-α* Tumor Necrosis Factor-alpha, *IL-6* Interleukin 6, *SAA* Serum Amyloid A, *COHb* carboxyhemoglobin

### Inflammation biomarkers

Decreases in inflammation biomarkers (Hs-CRP, TNF-α, IL-6, SAA, and COHb,) were found in the RAG compared with the PAG (Table 4).

### Correlation between nutritional hematological scores and inflammation biomarker parameters

Prealbumin was negatively correlated with Hs-CRP (*r* = − 0.41, *P* = 0.049), TNF-α (*r* = − 0.44, *P* = 0.042), IL-6 (*r* = − 0.54, *P* = 0.010), SAA (*r* = − 0.44, P = 0.042) and COHb (*r* = − 0.13, *P* = 0.318). Tf was negatively correlated with COHb (*r* = − 0.62, *P* = 0.001). Weight was negatively correlated with COHb (*r* = − 0.48, *P* = 0.0001), Hs-CRP (*r* = − 0.30, *P* = 0.18), TNF-α (*r* = − 0.44, *P* = 0.041), IL-6 (*r* = − 0.35, *P* = 0.11) and SAA (r = − 0.43, *P* = 0.046) (Table 5) (An additional figure file shows this in more detail (see Additional file 1, Additional file 2, Additional file 3, Additional file 4 and Additional file 5).

**Table 5:** Associations among the biomarkers at change from baseline to post treatment

	Weight	%IBW	Hb	Prealbumin	Tf	RBP
COHb	**− 0.48+**	**−0.48+**	− 0.25	−0.13	**− 0.62+**	−0.27
Hs-CRP	−0.30	−0.31	− 0.35	**−0.41+**	0.02	**−0.54+**
TNF-α	**−0.44+**	**−0.44+**	− 0.37	**−0.44+**	0.11	**−0.51+**
IL-6	−0.35	−0.36	**− 0.56+**	**−0.54+**	0.10	**−0.42+**
SAA	**−0.43+**	**−0.43+**	**− 0.55+**	**−0.44+**	0.13	−0.27

Significant associations (*p* < 0.05) are Bold and denoted with cross

*COHb* Carboxyhemoglobin, *Hs-CRP* High sensitivity C-reactive protein, *TNF-α* Tumor Necrosis Factor-alpha, *IL-6* Interleukin 6, *SAA* Serum Amyloid A, *%IBW* Percent Ideal Body Weight, *Hb* Hemoglobin, *Tf* Transferrin, *RBP* Retinol-Binding Protein

### Adverse reactions

The following minor adverse reactions were reported by some patients during the study: fatigue (four in the RAG and five in the PAG), subcutaneous hemorrhage (five in the RAG), dizziness (one in the RAG and two in the PAG), and needle site pain (five in the RAG). All events were minor reactions and patients recovered in a short time. No serious events due to acupuncture treatment were reported.

## Discussion

The present study is the first RCT on acupuncture treatments with precise evaluations of nutritional state and the BODE index of patients with COPD. Previously, we performed a study to evaluate the efficacy of acupuncture on nutritional state and the BODE index with accumulated COPD cases and found significantly improved prognosis in terms of the BODE index comparing before and after 10 weeks of acupuncture [19]. However, since there was no control group, the discussion whether acupuncture was capable of improving symptoms of COPD remained inconclusive.

In the present RCT study, we have found that there are many improvements in nutritional states of the RAG compared to those of the PAG. Above all, body weight increased 2.5 kg on average for the RAG. And there were no patients whose body weight increased in consequence of diseases such as cardiac incompetence or renal insufficiency during the 12 weeks of study period. Therefore, the increase in the body weight, we would say, was brought by improvements of nutritional status caused by acupuncture.

### Acupuncture effects on body composition and muscles strength in COPD

Weight loss is one of the main characteristics of advanced COPD, often associated with the increased susceptibility to exacerbations of respiratory symptoms, and considered to be an independent predictor of outcome [1]. Weight loss may involve all of the bodily tissue compartments, nevertheless, loss of skeletal muscle may be particularly important because wasting respiratory muscles leads to the loss of power and endurance [20]. Consequently, in physical examination, their measurements of arm muscle circumference (AMC) and triceps skin fold thickness (TSF) are significantly less than those of the healthy individuals [21].

In our study, although the circumferences of upper arm and thigh and the fat thicknesses of triceps skinfold and scapula skinfold increased significantly in the RAG compared to the PAG, the arm muscle circumference (AMC) which is a substitutional measurement of muscle mass did not show significant increase. Therefore, In the acupuncture group, weight gain of 2.5 kg on average was observed, and this weight gain was considered due to increase in fat mass rather than increase in muscle mass. In addition, it is presumed that the increase in fat mass accompanying acupuncture is related to improvement of appetite and reduction of inflammatory cytokines such as TNF-α and SAA.

At the same time, grip strength and maximum respiratory pressure improved significantly, which implies the recovery of muscle strength. Previous research reported that acupuncture stimulation has effects of relaxing muscle tension and relieving muscle fatigue [22]. Since many COPD patients have tension and fatigue in respiratory muscles and accessory respiratory muscles. Many of the acupuncture points used in this study are corresponding to accessory muscles of respiration. There are smaller pectoral muscle and superior posterior serratus muscle, which are the inspiratory accessory muscles, immediately under the LU1(Zhongfu) and the BL13(Feishu). Beneath the BL20(Pishu) and the BL23(Shenshu) there are inferior posterior serratus muscle and lumbar rectus muscle, which are the expiratory accessory muscles, and under the CV4(Guanyuan) and the CV12(Zhongwan), there is rectus abdominis muscle, which is also the expiratory accessory muscle. The rectus abdominis muscle and the lumbar rectus muscle work together to raise the abdominal pressure to make forced expiration and to hold breath. We considered that acupuncture to respiratory accessory muscles relaxed muscle tone and recovered muscle fatigue, which led to a significant improvement in respiratory muscle strength (MEP/MIP). Considering the contribution of nutritional status to improvement of muscle strength, we believe that acupuncture treatment had the combined effects to improve grip strength and respiratory muscle strength.

In addition, Takaoka et al. reported that when muscle atrophy was recovered by acupuncture in their animal experiment, lowered amount of expression of myostatin gene and increased number of muscle satellite cells were found. Myostatin gene has a function to suppress protein synthesis in muscle to prevent muscle mass to become excessive [23].

The suppression of myostatin gene expression leads to the activation of muscle satellite cells and the facilitation of muscle repair and muscular hypertrophy [24]. Therefore, we speculate that the lowered amount of myostatin gene expression caused by acupuncture led to the activation of muscle protein synthesis due to proliferation of muscle satellite cells; as a result, muscle atrophy was restored and muscle strength was recovered.

### Possible mechanism underlying the effect of acupuncture on malnutrition in COPD

Factors of malnutrition in COPD include anorexia and elevated metabolism which is reflected in the increased rest energy expenditure (REE), and factors of REE increase include decrease in ventilation efficiency due to obstructive ventilatory disorder and increase in respiratory muscle workload due to respiratory muscle fatigue [25]. Since patients with COPD often have lowered diaphragm due to hyperinflation of the lungs, intake of food into their stomach pushes up the diaphragm and diaphragmatic breathing is restricted and causes dyspnea. Therefore, such patients tend to avoid aggressive dietary intake and suffer nutritional disorders because of shortage of caloric intake, which leads to weight loss, skeletal muscle weakness, and respiratory muscle atrophy, resulting in easy fatigue of the skeletal muscle. Furthermore, since patients with COPD cannot take sufficient nutrition in spite of their significant energy consumption, they tend to fall into protein energy malnutrition (PEM) state. It has been also reported that the levels of transferrin and prealbumin, which are biomarkers of nutritional condition, of COPD patients are often low [26]. In our preceding study [9], it was found that many patients in the real acupuncture treatment group, compared to the placebo group, had improvement in dyspnea at the time of exertion, obstructive ventilatory impairment, accessory muscle of respiration fatigue and in addition, improvement in shortness of breath accompanying feeding behavior. It is considered that improvement of shortness of breath accompanying acupuncture treatment resulted in not only an increase in dietary intake, but also in a reduction of resting energy consumption which was increased by the disease. Consequently, we infer that improvements in nutritional status reflected in body weight were observed.

In addition, the main neural network that controls the feeding behavior is hypothalamus, but other parts such as paraventricular nucleus, arcuate nucleus, and solitary nucleus in medulla oblongata are also involved in the feeding regulation. ST36, the acupuncture point used in our study has been utilized from ancient times for disorders in gastrointestinal function.

Recent studies have found that acupuncture at ST36 sends stimulus to hypothalamus and solitary nucleus in medulla oblongata and efferently modulates peristalsis of digestive tract via vagus nerve [27, 28]. Therefore, in our study, we considered that acupuncture at ST36 affected the feeding center of the COPD patients and modulated peristalsis of digestive tract, which then led to the increase in the patients’ food intake and their better nutritional condition. Improvement in the gastrointestinal functions by acupuncture might have played a role in the increase of the biomarkers of nutritional condition in our trial.

Nutritional biomarkers utilized in our study are the ones generally used to monitor nutritional status of patients with chronic diseases since those biomarkers are sensitive enough to indicate a slight sign of malnutrition. In addition, since each biomarker has its own characteristic half-life period, such as 10–17 h for RBP, 2–4 days for prealbumin, and 7–10 days for transferrin, it is possible to make a tentative judgment on the duration of malnutrition. In our study, values of each biomarker have been significantly improved in the RAG compared to the PAG, therefore, we considered that the nutritional status has been improved at least 1 week before the examination.

Also, although the number of cases was rather small, it was recognized that inflammatory biomarkers had correlation with body weight and some nutritional biomarkers, therefore, we considered the possibility that the nutritional status was improved with the anti-inflammatory effect of acupuncture. On the other hand, interventional methods such as dietary advice and nutritional therapy for COPD patients, according to meta-analysis, have been found to significantly improve their body weight, fat free mass, maximum respiratory pressure, and grip strength. However, no significant improvements in FEV_1_, exercise tolerance, quadriceps muscle strength, or QOL were found [29, 30].

Currently, nutritional therapy including ω-3 fatty acids and ghrelin, which are of much attention recently, is recognized to increase body weight and muscle strength for COPD patients but there has been no fixed consensus on the detailed changes in nutrition and inflammatory biomarkers [31]. Furthermore, the prognosis of COPD patients who had nutritional therapy has not been studied.

Therefore, we consider that acupuncture can be, as our research results indicate, one of the useful methods other than nutritional therapy to improve the nutritional status of COPD patients.

### Acupuncture impacts on inflammation in COPD

In our study, inflammatory material or inflammatory cytokine such as COHb, Hs-CRP, SAA, IL-6, TNF-α decreased in the RAG compared to those in the PAG.

It has been reported that unexplained weight loss (muscle wasting and adipose tissue depletion), which is a characteristic feature of advanced COPD, is linked to the systemic inflammation [8]. And bodyweight loss has been linked to the reduction of CRP, TNF-α, IL-1β or IL-6 in most previous studies which considered systemic inflammation in COPD [5]. It has been reported that the functional disorder in skeletal muscle is usually found in COPD due to the wasting of skeletal muscle and change in its quality and the increased level of TNF-α, IL-6, and CRP is related to the lowered muscular strength [32, 33].

Models of anti-inflammatory effects of acupuncture were reviewed by John et al. and summarized to several physiological pathways: hypothalamus-pituitary-adrenal (HPA) axis, sympathetic pathways (via both sympathetic postganglionic neurons and the sympathoadrenal medullary axis), parasympathetic cholinergic pathways, antihistamine effects, down regulation of proinflammatory cytokines (such as TNF-α, IL-1β, IL-6, and IL-10), and suppression of the expression of COX-1, COX-2, and iNOS [34].

The acupuncture points used in this study (CV12(Zhongwanh), ST36(Zusanli), BL20(Pishu)) are traditionally recognized to be effective for the symptoms of the digestive system. ST36, especially, has been used in many basic researches and is the acupuncture point with a lot of clinically accepted evidences. Recent studies have shown that acupuncture stimulation at ST36 could regulate the nerve-endocrine-immune network [35]. Acupuncture stimulation at ST36 can regulate a wide variety of diseases caused by inflammation, and can also modulate TNF-α, IL-1β and IL-6 levels [36, 37].

Also, SAA is an acute phase reactant which corresponds to IL-1, IL-6, TNF-α, etc., and is an amyloidogenic protein, therefore, it is considered that SAA is decreased since IL-6 and TNF-α in the RAG are significantly decreased.

Furthermore, in chronic inflammatory diseases, it is often found that hemoglobin level is low, like in anemia, our study showed that hemoglobin level was improved in the RAG compared to the PAG. John et al. found that erythropoietin level is significantly higher in COPD patients with anemia compared to those without anemia [38]. Also, in COPD patients with anemia, hemoglobin level and erythropoietin level are inversely correlated, which suggests a decrease in hematopoietic response to erythropoietin. Such decrease in hematopoietic response to erythropoietin can be inferred to be caused by increase of inflammatory cytokines such as TNF-α, IL-6, and INF-γ [39]. Consequently, the improvement of hemoglobin level in RAG found in our study implies that the anti-inflammatory effect of acupuncture decreased inflammatory mediators.

### Prognosis

Nutritional status is an important determinant of symptoms, disability, and prognosis in COPD, and being underweight can be problematic. The reduction in BMI is an independent risk factor for mortality of COPD patients.

The mean reduction of the BODE index of COPD patients was significantly greater in the RAG than that in the PAG. Our study clearly demonstrated that BMI, %FEV_1_, MRC criteria and 6MWD, within the BODE index, were improved in COPD patients after 12 weeks of acupuncture [9].

Celli et al. reported that the minimal difference which is clinically important for the BODE index is 1 U or more [40]. In the present study, therefore, the effect of acupuncture on the BODE index was satisfactory great. It is conceivable that acupuncture may affect prognosis of COPD patients since the acupuncture treatment improved all factors included in the BODE index. This result suggests that acupuncture may have a considerable influence on the prognosis of COPD patients, especially for those with advanced disease.

### Study limitation

Patients recruited in the present study did not have any supplement or vitamin newly prescribed by medical facilities for 6 months prior to the study. However, two patients in the PAG had been continually prescribed and used a supplement for at least 2 years prior to the study and five patients in the PAG and two patients in the RAG had personally purchased from drugstores and used multivitamins for at least 3 years prior to the study. We considered this would cause no effects on our study since all of them had been taking those supplements or vitamins for over the years and did not change their habits.

This study was performed in 4 different facilities. At one of these facilities, nutritional evaluation (Transferin, RBP) could not be obtained, and at two of them, inflammatory evaluation could not be assessed. Also, the number of blood sample was not large enough. In addition, since we did not record the amount of food intake and meal content of all patients, the details about changes in calorie intake and diet were unknown.

## Conclusion

We demonstrated clinically relevant improvements in nutritional states (body composition, and nutritional hematological examination), prognosis (BODE index), muscle strength and inflammation biomarkers in COPD patients after 3 months of acupuncture treatment in our RCT. In order to clearly confirm the usefulness of acupuncture as an adjunctive therapy in COPD treatment, randomized trials with larger sample sizes and longer-term interventions with follow up evaluations are necessary.

## Additional files

Additional file 1: Correlation between COHb and Prealbumin is shown. Prealbumin was negatively correlated with COHb (*r* = − 0.13, *P* = 0.318), (Spearman’s rank correlation coefficient). COHb; carboxyhemoglobin. Cross:Placebo Acupuncture Group, Open circle; Real Acupuncture Group. (PPTX 43 kb)

Additional file 2: Prealbumin was negatively correlated with Hs-CRP (*r* = − 0.41, *P* = 0.049). (Spearman’s rank correlation coefficient). Hs-CRP; High sensitivity C-reactive protein. Cross:Placebo Acupuncture Group, Open circle; Real Acupuncture Group. (PPTX 42 kb)

Additional file 3: Prealbumin was negatively correlated with TNF-α (*r* = − 0.44, *P* = 0.042). (Spearman’s rank correlation coefficient). TNF-α; Tumor Necrosis Factor-alpha. Cross:Placebo Acupuncture Group, Open circle; Real Acupuncture Group. (PPTX 42 kb)

Additional file 4: Prealbumin was negatively correlated with IL-6 (*r* = − 0.54, *P* = 0.010). (Spearman’s rank correlation coefficient). IL-6; Interleukin 6. Cross:Placebo Acupuncture Group, Open circle; Real Acupuncture Group. (PPTX 42 kb)

Additional file 5: Prealbumin was negatively correlated with SAA (*r* = − 0.44, P = 0.042). (Spearman’s rank correlation coefficient). SAA; Serum Amyloid A. Cross:Placebo Acupuncture Group, Open circle; Real Acupuncture Group. (PPTX 41 kb)

## Abbreviations

%IBW: Percent ideal body weight
6MWD: 6-min walking distance
ANCOVA: analysis of covariance
BMI: Body mass index
CAT: COPD acupuncture trial
CI: confidence intervals
COHb: Carboxyhemoglobin
COPD: Chronic obstructive pulmonary disease
COX: Cyclooxygenase
CRP: C-reactive protein
FEV_1_: Forced expiratory volume in one second
GOLD: Global initiative for chronic obstructive lung disease
Hb: Hemoglobin
HPA: Hypothalamus-pituitary-adrenal
H-s-CRP: High sensitivity C-reactive protein
IL-1β: Interleukin 1beta
IL-6: Interleukin 6
INF-γ: Interferon-gamma
iNOS: inducible-Nitric oxide synthase
MAC: Midupper arm circumference
MEP: maximum expiratory mouth pressure
MIP: Maximum inspiratory mouth pressure
MRC: Medical research council
PA: Pre-albumin
PAG: Placebo acupuncture group
QOL: Quality of Life
RAG: Real acupuncture group
RBP: Retinol-binding protein
REE: Rest energy expenditure
SAA: Serum amyloid A
SSF: Scapula skin folds (SSF)
STF: Subscapular skinfold thickness
Tf: Transferrin
TNF-α: Tumor necrosis factor-alpha
