# Supplementary figures and images for: Effects of acupuncture on nutritional state of patients with stable chronic obstructive pulmonary disease (COPD): re-analysis of COPD acupuncture trial, a randomized controlled trial
Source: BMC Complement Altern Med. 2018 Oct 24;18:287. doi: 10.1186/s12906-018-2341-3 (PMC6201549; doi:10.1186/s12906-018-2341-3)

## Slide 1
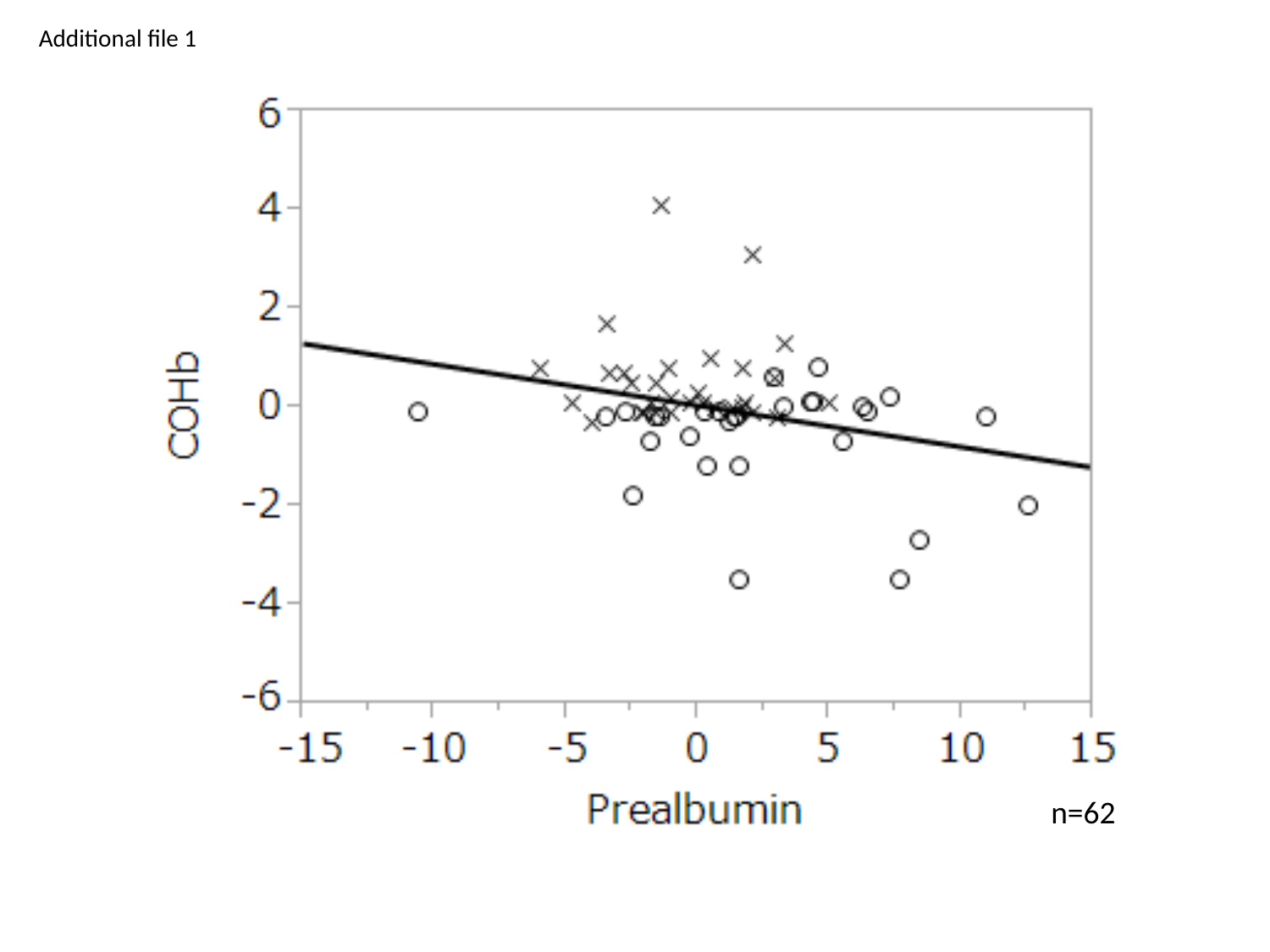

Additional file 1
n=62

Supplement: Supplementary file 1 — Correlation between COHb and Prealbumin is shown. Prealbumin was negatively correlated with COHb (r = − 0.13, P = 0.318), (Spearman’s rank correlation coefficient). COHb; carboxyhemoglobin. Cross:Placebo Acupuncture Group, Open circle; Real Acupuncture Group. (PPTX 43 kb) [file 12906_2018_2341_MOESM1_ESM.pptx]

## Slide 1
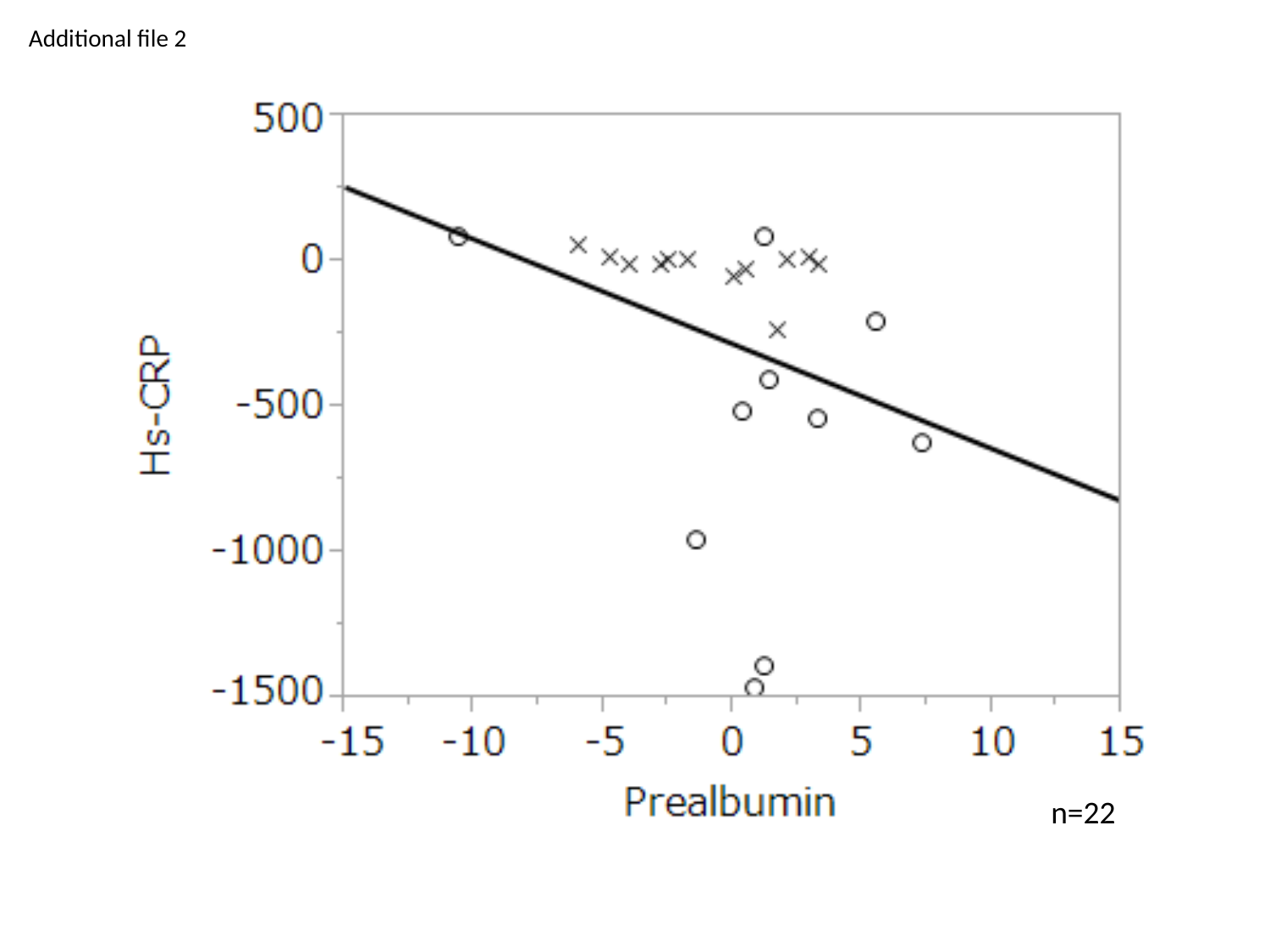

Additional file 2
n=22

Supplement: Supplementary file 2 — Prealbumin was negatively correlated with Hs-CRP (r = − 0.41, P = 0.049). (Spearman’s rank correlation coefficient). Hs-CRP; High sensitivity C-reactive protein. Cross:Placebo Acupuncture Group, Open circle; Real Acupuncture Group. (PPTX 42 kb) [file 12906_2018_2341_MOESM2_ESM.pptx]

## Slide 1
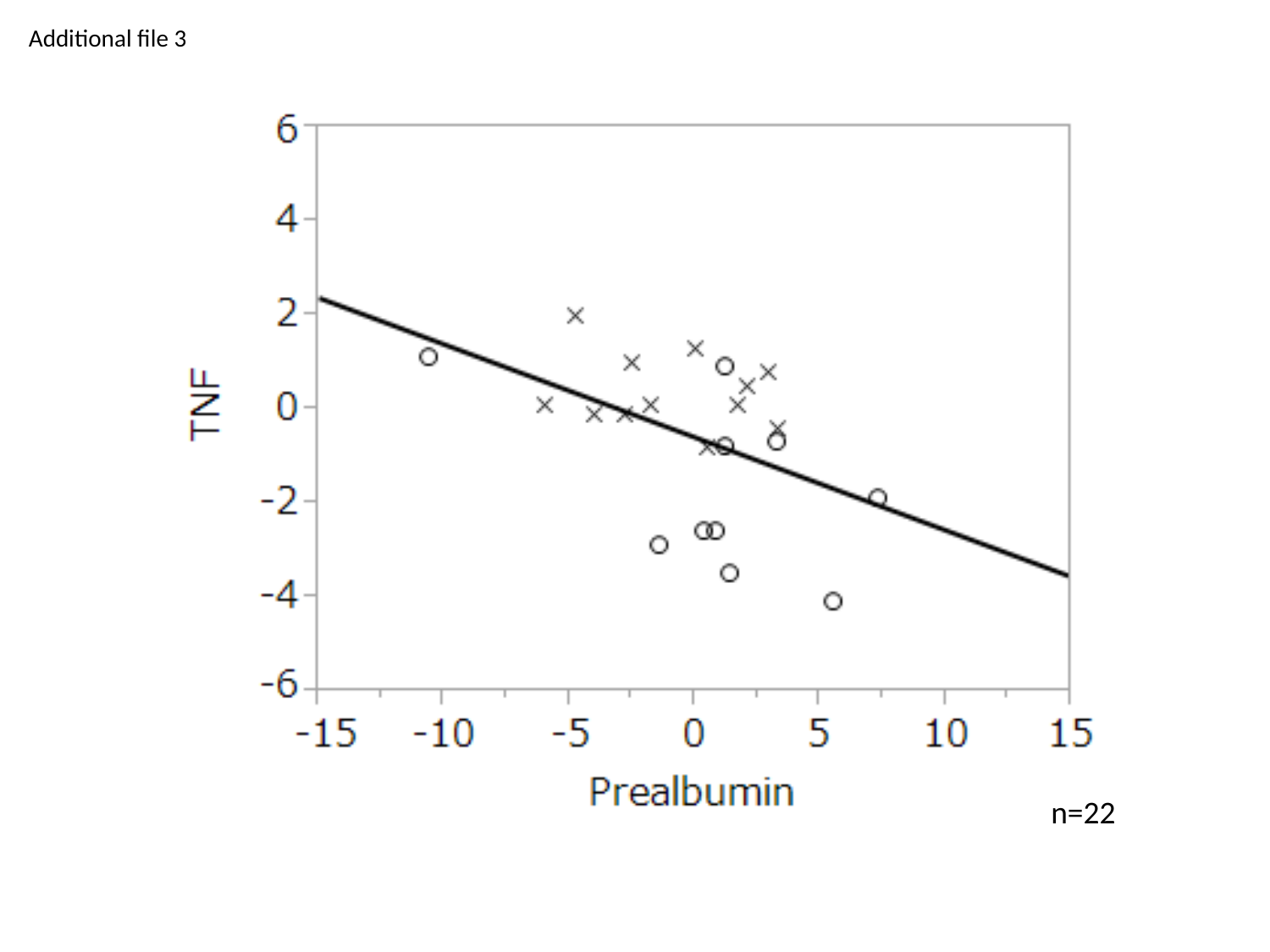

Additional file 3
n=22

Supplement: Supplementary file 3 — Prealbumin was negatively correlated with TNF-α (r = − 0.44, P = 0.042). (Spearman’s rank correlation coefficient). TNF-α; Tumor Necrosis Factor-alpha. Cross:Placebo Acupuncture Group, Open circle; Real Acupuncture Group. (PPTX 42 kb) [file 12906_2018_2341_MOESM3_ESM.pptx]

## Slide 1
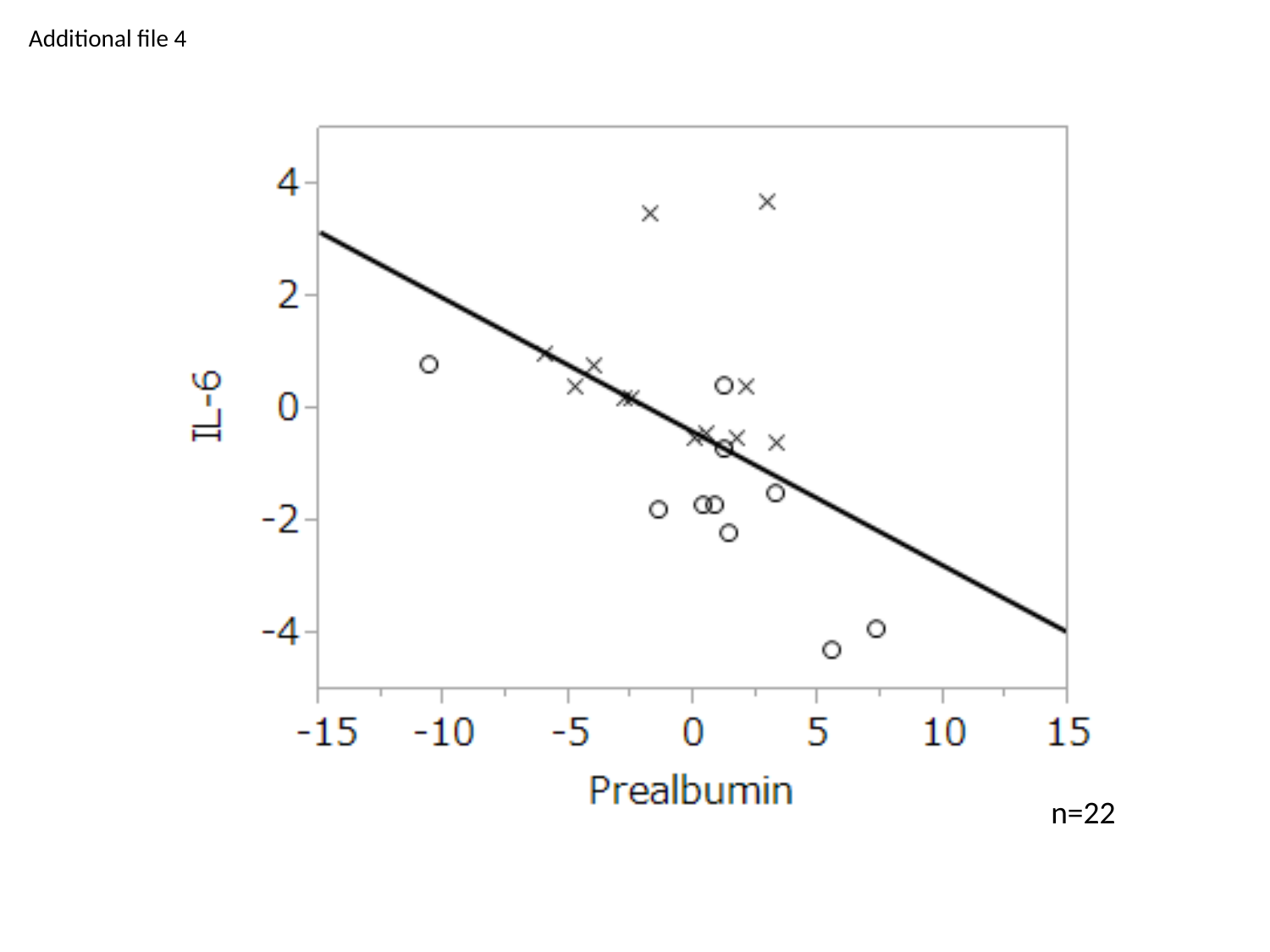

Additional file 4
n=22

Supplement: Supplementary file 4 — Prealbumin was negatively correlated with IL-6 (r = − 0.54, P = 0.010). (Spearman’s rank correlation coefficient). IL-6; Interleukin 6. Cross:Placebo Acupuncture Group, Open circle; Real Acupuncture Group. (PPTX 42 kb) [file 12906_2018_2341_MOESM4_ESM.pptx]

## Slide 1
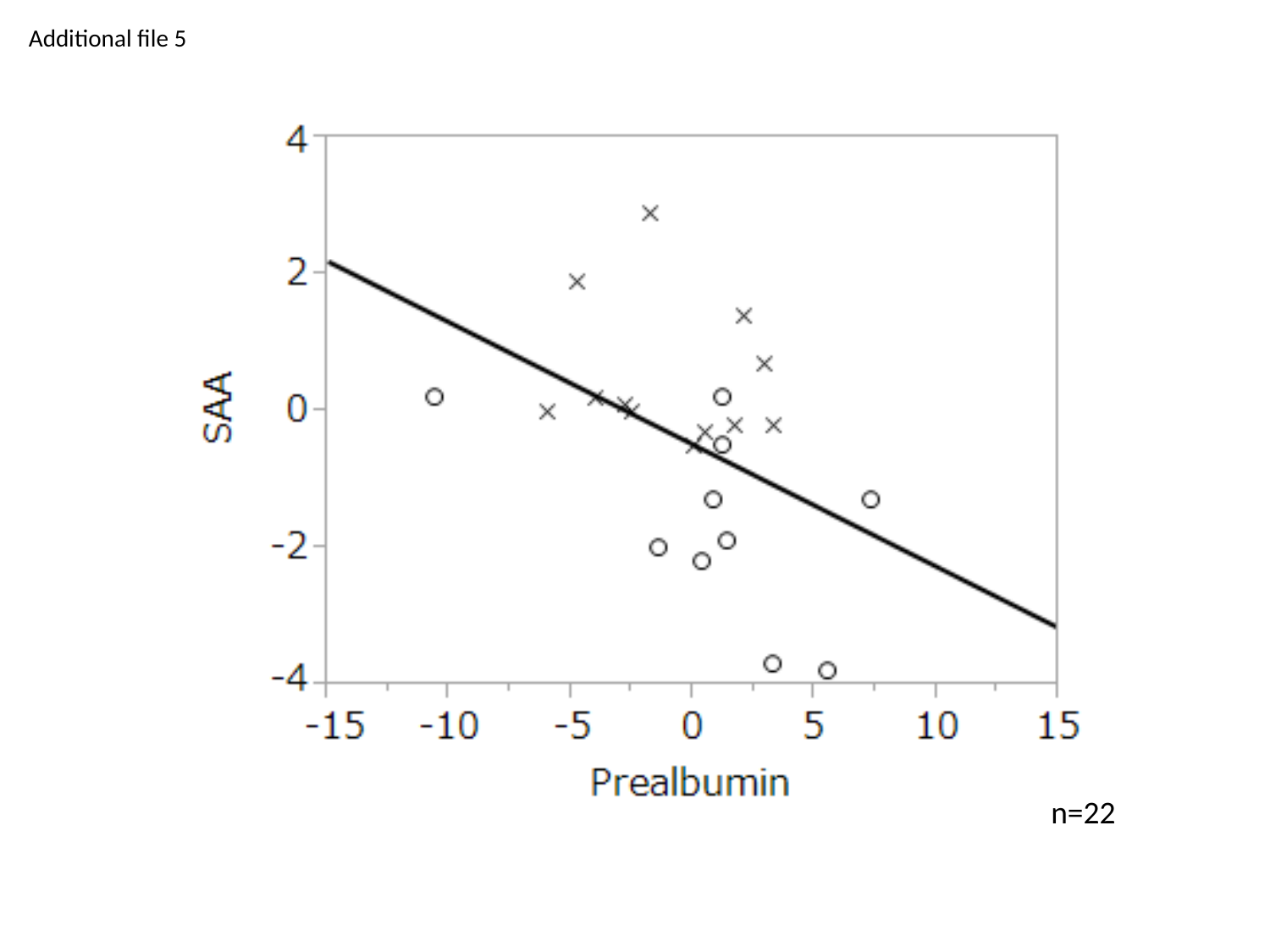

Additional file 5
n=22

Supplement: Supplementary file 5 — Prealbumin was negatively correlated with SAA (r = − 0.44, P = 0.042). (Spearman’s rank correlation coefficient). SAA; Serum Amyloid A. Cross:Placebo Acupuncture Group, Open circle; Real Acupuncture Group. (PPTX 41 kb) [file 12906_2018_2341_MOESM5_ESM.pptx]
